# Seasonal variation in aquatic habitat availability and use by the malaria vector, *Anopheles funestus*

**DOI:** 10.1186/s12936-026-05898-w

**Published:** 2026-04-11

**Authors:** Najat F. Kahamba, Khamisi Kifungo, Mohammed Jumanne, Fedra Trujillano, Marceline F. Finda, Betwel Msugupakulya, Francesco Baldini, Luca Nelli, Fredros O. Okumu, Heather M. Ferguson

**Affiliations:** 1 Environmental Health and Ecological Sciences Department, Ifakara Health Institute, P. O. Box 53, Ifakara, Tanzania; 2 School of Biodiversity One Health and Veterinary Medicine, University of Glasgow, Glasgow, UK; 3 School of Public Health, Faculty of Health Science, University of the Witwatersrand, Johannesburg, South Africa; 4 School of Life Science and Biotechnology, Nelson Mandela African Institution of Science and Technology, P. O. Box 447, Arusha, Tanzania

**Keywords:** *Anopheles funestus*, Malaria, Aquatic habitats, Seasonal variation, Remote sensing, Larval source management, Kilombero Valley, Tanzania

## Abstract

**Background:**

Larval source management (LSM) can be highly effective for controlling malaria vectors such as *Anopheles funestus s.s.*, which typically exploit large and permanent aquatic habitats. While these habitats can persist year-round in endemic regions of Africa, their availability and use shift between wet and dry seasons. Understanding these seasonal changes is essential for identifying the habitats that sustain vector populations and for determining when and where LSM would be most effective.

**Methods:**

We investigated the availability and use of *An. funestus* larval habitats across wet and dry seasons in south-eastern Tanzania, and the environmental factors that influence these patterns. Cross-sectional surveys were conducted in five villages during the dry season (September–November 2021) and rainy season (February–May 2022) to map and characterize aquatic habitats and identify those colonized by *An. funestus*.

**Results:**

In total, 2824 aquatic habitats were identified, of which 27% were positive for *An. funestus*. Remotely sensed land cover data and directly measured habitat characteristics were incorporated into generalized linear mixed models to evaluate seasonal and environmental predictors of larval presence and abundance. Larval occurrence and density were significantly influenced by habitat type, village, season, and their interactions, as well as by key physicochemical factors including water depth, vegetation type, algae, water clarity, and water source. *An. funestus* was commonly found in river streams, ground pools, and ditches across both seasons. During the wet season, however, it also occupied spring-fed wells, rice fields, and dug pits, indicating broader habitat use.

**Conclusion:**

These findings demonstrate a clear seasonal shift in larval habitat use by *An. funestus*, reflecting its ecological adaptability. While the species generally favors permanent habitats, its expanded use of diverse sites in the wet season has important implications for LSM. Targeting persistent habitats during the dry season may offer a more efficient and feasible window for implementing this intervention.

**Supplementary Information:**

The online version contains supplementary material available at 10.1186/s12936-026-05898-w.

## Introduction

Understanding how animals shift their habitat selection in response to changing environmental conditions is a core question in ecology, with implications for predicting species distributions and responses to environmental change. This question is particularly important for pest species such as insect vectors, where environmental management strategies depend on knowing when and where target organisms utilize specific habitats [45]. In the case of malaria-transmitting mosquitoes, understanding the relationship between the availability of aquatic habitats and the dynamics of vector populations is essential for designing effective control interventions [49].

The diversity and availability of aquatic habitats used for larval development by African malaria vectors undergo significant seasonal variation due to rainfall [27, 35]. For instance, most malaria-endemic regions of Africa experience at least one prolonged ‘dry’ and ‘wet’ season per year. During the dry season, only large, more permanent water bodies such as ponds and rivers may be prevalent [20], while temporary habitats like puddles and ditches proliferate during the rains [29, 30]. These changes in habitat distribution and availability impact the spatial and temporal distribution of adult mosquito populations [18], which are key to managing malaria transmission dynamics. Understanding how these fluctuations in habitat availability translate into use by malaria vectors is crucial both for understanding the dynamics of their populations, and effective targeting of vector control strategies [19].

Seasonal variation in rainfall leads to both substantial variation in water availability and land cover in African settings [16, 42, 44]. While presence of suitable aquatic habitats is essential for vector population persistence, there can be substantial variation between the use (occupancy) and abundance of larvae in different aquatic habitats in relation to their physico-chemical properties such as temperature, nutrient levels, water movement, and presence of predators or competitors [7, 20]. Additionally, there can be substantial variation in the use of aquatic habitats between mosquito species. For example, the major African malaria vector *An. gambiae s.l.,* can use diverse aquatic habitats including puddles, rice fields, and hoofprints [25, 34]. In contrast *An. funestus s.l.,* another very important vector species, tends to occupy large more permanent water bodies with vegetation [8, 20], Ismail H. [36, 37]. These ecological distinctions underline the need to understand whether and how *An. funestus* adjusts its habitat use across seasons, a question central to this study.

While the effect of seasonality on mosquito vector abundance has been extensively studied [20, 27], much less is known about how mosquitoes adapt their use of larval habitats in response to seasonal changes in habitat availability. Understanding how malaria vectors adapt their selection and use of aquatic habitats across the year is essential for guiding the design and implementation of effective Larval Source Management (LSM) control strategies that are optimally timed and targeted.

Despite the recognized importance in driving malaria vector dynamics, there is still a considerable knowledge gap regarding the impact of seasonality on the quantity, distribution and use of aquatic habitats by malaria vectors. This is particularly true for *An. funestus,* the most important vector of malaria in southern Tanzania and several other settings in east and southern Africa [33]. While there has been extensive research on the larval ecology of other primary vectors like *An. gambiae s.s* and *An. arabiensis* [27, 28, 43], *An. funestus* has received much less attention [19, 38]. The distinct ecology of this species means its seasonal habitat use is unlikely to be generalizable from other African vectors. Specifically, in contrast to other malaria vectors that use temporary aquatic habitats (e.g., puddles [28], *An. funestus* larvae are often found in larger, more permanent water bodies [7, 20], Ismail H [36, 37]. As a result, *An. funestus* has been proposed as a good candidate for LSM on account of the World Health Organization’s criteria of having larval habitats that are “fixed, few, and findable” [24, 49, 50]. However, it is hypothesized that *An. funestus* might adapt its larval habitat use seasonally in response to availability, including possible expansion into temporary and or harder to reach habitats during the rainy season. Such seasonal adaptation in larval habitat use would increase the complexity of LSM and warrant careful consideration of the timing and selection of habitats for optimal impact.

Here we investigated seasonal variation in the availability and environmental characteristics of aquatic habitats available to *An. funestus* and tested how this influenced their habitat use. The study was conducted in south-eastern Tanzania where *An. funestus* is the predominant malaria vector [21]. Specific objectives were: (i) to estimate seasonal variation in the availability and type of aquatic habitats, (ii) assess how the types of aquatic habitats used by *An. funestus,* and larval abundance varies seasonally; and (iii) examine the spatial distribution of aquatic habitats across seasons. Results will be of value for informing LSM strategies for improving malaria control initiatives, particularly in areas where *An. funestus* is the dominant malaria vector.

## Methods

### Study area

**Fig. 1:**
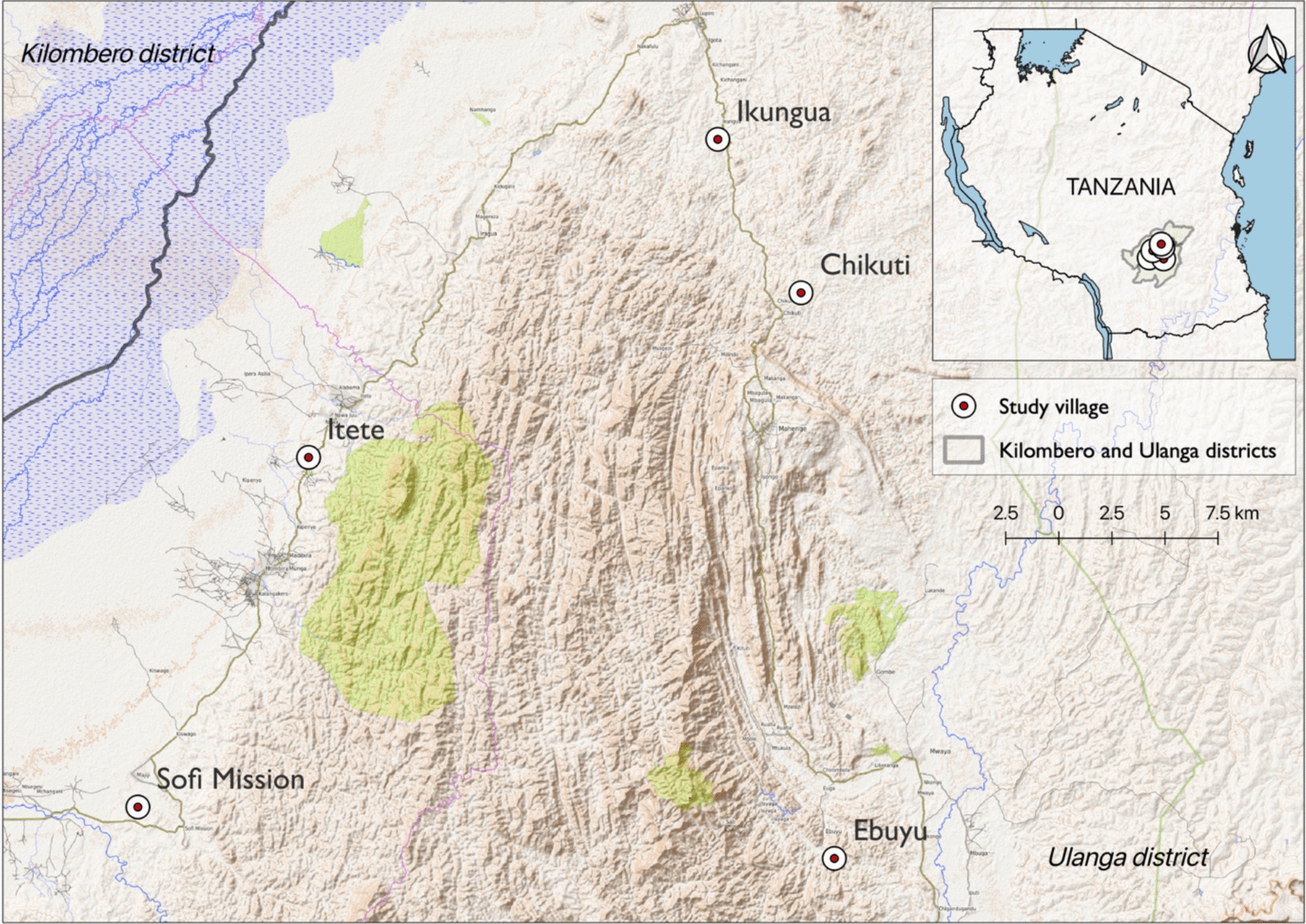
Showing the geographic locations of the five study villages (red circles) within Ulanga districts, in south-eastern Tanzania

The study was conducted in five villages within the districts of Ulanga and Malinyi in south-eastern Tanzania: Ikungua (− 8.4633°, 36.6873°), Chikuti (− 8.6028°, 36.7288°), Ebuyu (− 8.9719°, 36.7608°), Itete (− 8.7202°, 36.3435°), and Sofi Majiji (− 8.9278°, 36.2682°) approximately 100 km from Ifakara town (Fig. 1). These villages were selected based on known malaria prevalence [32], presence of *An. funestus* [20] and varying altitude (high: > 400 m, and low =  < 200 m). Although some supporting references were published after data collection, village selection was based on long-established malaria transmission patterns, prior entomological surveillance, and local programmatic knowledge available at the time of study design. There is marked seasonal variation in temperature and rainfall across the study area; with the dry season typically running from June to November and highest temperatures falling between September and November. There are two distinct wet seasons: the short (December–January) and longer rainy season (February–May). On average the monthly temperatures ranged between 20 °C and 32.6 °C and average annual rainfall was ~ 1500 mm.

### Study design

A repeated cross-sectional survey was conducted to assess the distribution of aquatic habitats and the presence and abundance of *An. funestus* larvae during the dry (September–November 2021) and rainy season (February–May 2022). Comprehensive surveys of all aquatic habitats were conducted in each study village in each season. The mapping of habitats involved a structured approach [20], where transects were systematically walked across the area of each village to identify all potential aquatic habitats ranging from large, permanent water bodies such as ponds and river streams to small temporary habitats like puddles, ditches, and hoofprints.

### Characterization of aquatic habitats and larval sampling

The exact locations of all identified aquatic habitats were recorded using a hand-held Garmin eTrex 30 × GPS GPS receiver. Different physico-chemical characteristics of each habitat were measured and recorded (SOM Table 1). Surveys were conducted in all potential habitats to assess the presence and abundance of mosquito larvae. Here a water body, irrespective of the presence of larvae, was defined as an “aquatic habitat,” whereas "larval habitat" refers to aquatic habitats where larvae were detected. Habitats with at least one *An. funestus* larva were classified as *An. funestus* habitats. Depending on the size and depth of aquatic habitats, larval surveys were performed using a standard 350 ml dipper or a 10-L bucket. Specifically, the 10-L bucket was employed for larger habitats, typically those with water depths exceeding 30 cm (SOM Fig. 1). To quantify the larvae, samples from each dip (standard dipper or 10-L buckets) were counted and recorded [20]. The contents of the dipper or bucket were carefully poured or filtered into a white tray for subsequent counting and sorting. For standardization, we defined a new variable named *‘Total volume sampled (TVS)’,* calculated using the following formula:

$$TVS=\left\{\begin{array}{c}Number of dips\times 10 if sampling method is 10l Bucket\\ Number of dips\times 0.35 if sampling method is 350ml dipper\end{array}\right.$$

This formula calculated the total volume of water sampled based on the sampling method. This variable was used to control for variation in sampling effort when analyzing mosquito presence and abundance as described in analysis methods. All collected mosquito larvae were morphologically identified in the field using established taxonomic keys by Gillies and De Meillon and Gillies and Coetzee [14, 15]. Larval identification was conducted by trained entomology technicians from the Ifakara Health Institute who had received formal and standardized training in mosquito larval identification prior to the surveys. Larvae were identified into taxonomic groups of *An. funestus *sensu lato (*sl*), *An. gambiae sl*, and *Culex*, and others. Acquisition of remote sensing data.

Satellite observation data were extracted from Planet Explorer platform, which provides data from the PlanetScope commercial satellites [13]. These satellites are known for their high spatial (3 m) and temporal (daily) resolution [41]. Images were selected to align with sampling periods in the dry (August–November 2022) and rainy (February–May 2022) seasons. Priority was given to images with the best image quality and minimal cloud coverage within the specified duration. The PlanetScope optical data consists of eight spectral bands: blue, green, red, near-infrared (NIR), coastal blue, green I, yellow, and red edge [13, 41]. We used the green and NIR bands to calculate Normalized Difference Water Index (NDWI), as this is appropriate for detecting water [12].

### Data analysis

All analyses were done using R statistical software version 3.7.1 [40].

#### Seasonal variation availability of aquatic habitats

Seasonal variation in aquatic habitat availability was estimated in two ways. First, PlanetScope data was used to assess seasonal shifts in the proportion of land covered by water. We employed a classification algorithm to categorize land into water and non-water areas using multispectral image data. In the *R* environment, training data for the classifier were generated through random sampling within water and non-water polygons, identified from satellite images and validated on the ground, producing 1000 points for each class. These points were then used to extract corresponding spectral band values from the multispectral images. A recursive partitioning decision tree model (*rpart* package) was employed to develop the classification algorithm [46]. The model used the extracted band values as predictors for the binary classification task. The decision tree was visualized to ensure interpretability of the classification criteria. Model performance was evaluated using a confusion matrix, with accuracy and the Kappa statistic as key metrics. The confusion matrix was visualized using a custom *ggplot2* function, *ggplotConfusionMatrix*, which highlighted the proportion of correct and incorrect classifications within the dataset [26]. Classifications from this model were used to indirectly measure seasonal changes in water availability based on proportion of land classified as water. Additionally, seasonal variation in aquatic habitat availability was directly estimated from field observations by comparing the quantity and types of aquatic habitats recorded in each season.

#### Seasonal and environmental determinants of the presence and abundance of *An. funestus* larvae in the different aquatic habitats

Aquatic habitat used by *An. funestus* was defined in terms of positivity (% habitats in which *An. funestus* larvae were present) and productivity (mean abundance of *An. funestus* larvae per dip across different habitats). First, the number and types of aquatic habitats used by *An. funestus* larvae in different seasons were tabulated for descriptive analysis. Second, statistical models were built to test for variation in *An. funestus* larval positivity and abundance as described below.

Generalized Linear Mixed Models (GLMM) were employed to estimate predicted mean larval positivity and abundance in habitats, and how they varied between seasons, habitat types and in association with physico-chemical characteristics [2]. For positivity analysis habitats were coded '0' for *An. funestus* absence, and ‘1’ for presence. Given the primary emphasis on investigating seasonality, covariates were included season (rainy or dry), its interaction with habitat type, and environmental factors known to affect *An. funestus* larval ecology such as, vegetation quantity, water clarity, and water source (see SOM Table 2 Model 2 for full details). The interaction between season and habitat type was specifically fit to allow testing of the hypothesis that the types of larval habitats used by *An. funestus* varied between seasons. Random effects for sampling date were included to account for potential dependencies in observations.

Variations in larval abundance were analyzed using a GLMM with a zero-inflated negative binomial distribution (ZINB) to address data overdispersion and excess of 0 s [1]. Overdispersion was evident from variance exceeding the mean, and excess zeros reflected true absence of larvae rather than sampling effects. Like the GLMM for analysis of positivity, the model integrated fixed effects including habitat type, village, vegetation cover, and algae type, along with random effects for sampling date (SOM Table 2, Model 3).

Both models shared the same set of explanatory variables, and model selection was performed using likelihood ratio tests (LRTs) via stepwise backward selection. Biologically plausible interactions were explored during model selection, with the season × habitat type interaction retained, sampling date was included as a random effect. Standardized total volume of water sampled was included as an offset term to normalize sampling efforts differences.

#### Spatial distribution of habitats in wet and dry season

The spatial distribution of larval habitats was analyzed to examine the location and clustering of *An. funestus* larvae throughout the study area, and how they varied seasonally. *Moran's* I statistic was calculated within the *spdep* package in R to summarize the degree of spatial autocorrelation in An. *funestus* positive habitats [3]. A *Moran's* I value close to + 1 indicates strong clustering of habitats, whereas a value near -1 suggests dispersion. To complement this, we employed Inverse Distance Weighting (IDW) interpolation using the *gstat* package in R to estimate the spatial distribution patterns of *An. funestus* habitats in areas that were not sampled, based on the proximity to sampled points. This method assigns more weight to nearer points, implying that habitats closer to sampled locations have a greater influence on the interpolation result. For our analysis, we used a 2-m distance from the sampled points to estimate the weight of neighboring points. A high IDW value indicates a high likelihood of habitat occurrence in each area, which is important for understanding and predicting larval habitat distribution.

## Results

### Seasonal variation in the availability and types of aquatic habitats

High-quality satellite image data with minimal cloud coverage was successfully obtained for four of the five study sites: Ikungua, Sofi Majiji, Ebuyu, and Itete. The total area of land covered by water contracted by 40% between the wet (121.64 km^2^) and dry season (72.92 km^2^). The magnitude of change was consistent across villages; with Sofi Majiji and Ebuyu experiencing the greatest change between wet and dry season (approximately halving, Table 1).

**Table 1:** Seasonal changes in water coverage by village

Village		Rainy season		Dry season	
	Surface Area (km^2^)	Area covered by water (km^2^, %)	Accuracy %, (Kappa statistics)	Area covered by water (km^2^, %)	Accuracy %, (Kappa statistics)
Ikungua (Ulanga district)	60.40	36.4 (60.31%)	87, (0.73)	27.8 (46.14%)	92, (0.84)
Ebuyu (Ulanga district)	61.99	35.09 (56.59%)	59, (0.13)	16.7 (27.03%)	93, (0.85)
Sofi Majiji (Malinyi district)	72.12	26.56 (36.83%)	85, (0.71)	13.3 (18.31%)	88, (0.77)
Itete (Malinyi district)	50.64	23.59 (46.59%)	94, (0.87)	15.12 (29.86%)	90, (0.81)
Totals	245.15	121.64		72.92	

*This table presents the data for dry and rainy seasons in the four villages, including total surface area, percentage water area, and accuracy along with Kappa statistics for the water classification*

Mirroring trends predicted from remote sensing, the total number of observed aquatic habitats varied significantly across the seasons, from 2,485 in the wet season to 339 in the dry season (Table 2). Some habitat types experienced larger seasonal declines than others. For example, between the wet and dry season, the number of stream segments decreased from 950 to 193, ditches from 499 to 5 and ground pools from 122 to 26. This resulted in a reduction of habitat diversity in the dry season; with, 9 different types present in the wet season (and ~ 100 or more each of each type), compared to only 7 habitat types in the dry season (all in lower numbers, with only the ‘river and stream segment’ category having more than 100 habitats). Rice field and tire track habitats were relatively common in the wet season but were absent during the dry season (Table 2).

**Table 2:** Total habitats, positivity, and abundance for *An. funestus* in dry rain and season

Habitat type	Rainy season			Dry season		
	Total habitats (N)	Habitats positive for *An. funestus* (%)	Mean abundance of *An. funestus/* dip	Total habitats (N)	Habitats positive for *An. funestus* (%)	Mean abundance of *An. funestus/* dip
River/Stream segments	950	295 (31)	0.55	193	133 (69)	1.51
Ground pools	122	46 (38)	0.58	26	11 (42)	1.27
Ditches	499	140 (28)	0.32	5	3 (60)	2.32
Dug pits & holes	318	20 (6)	0.28	24	0 (0)	0
Spring fed wells	186	64 (34)	1.69	71	4 (5)	0.02
Brick & concrete pits	135	8 (6)	0.24	18	0 (0)	0
Rice fields	176	34 (19)	0.06	0	0 (0)	0
Tire track	95	3 (3)	0.03	0	0 (0)	0
Hoofprints	4	0 (0)	0	2	0 (0)	0
Totals	**2485**	**610 (24.5)**	**-**	**339**	**151 (44.5)**	**-**

*This table summarizes the overall number of all habitats identified, the positive and proportion of An. funestus and their mean abundance across the season*

### Mosquito species composition and abundance

A total of 69,241 immature mosquitoes, including larvae and pupae, were collected from aquatic habitats in this study. Substantially more larvae were collected during the rainy (56,058) than dry season (13,062), reflecting the expansion of aquatic habitats (Table 3). The sampling effort involved 4869 dips in the dry season and 33,285 dips in wet season. During the dry season, the predominant *Anopheline* species was *An. funestus s.l*, (37% of all mosquito larvae, n = 4840), followed by *An. gambiae s.l* (16%, n = 2067), other *Anopheline* species (8%) and *Culicine* mosquitoes (39%). In the rainy season, *An. funestus* constituted only 20% of the mosquito larval community, with *An. gambiae sl* being the dominant Anopheline species, (32% of mosquito community, Table 3).

**Table 3:** Mosquito larvae collected in dry and wet seasons

Habitat Type	Rainy season					Dry season				
	Count	*An. funestus*	*An. gambiae s.l*	Other *Anopheles*	*Culex*	Count	*An. funestus,*	*An. gambiae s.l*,	Other *Anopheles*	*Culex*
	(N)	n (%)	n (%)	n (%)	n (%)	(N)	n (%)	n (%)	n (%)	n (%)
River streams	20,235	6976 (34)	4604 (23)	1733 (9)	6,922 (34)	10,536	4502 (43)	1150 (11)	889 (8)	3,995 (38)
Ground pools	6,229	971 (16)	1987 (32)	781 (13)	2,490 (40)	1391	256 (18)	544 (39)	94 (7)	497 (36)
Ditch	8,749	1719 (20)	3154 (36)	1357 (16)	2519 (29)	134	52 (39)	5 (4)	27 (20)	50 (37)
Dug pits & holes	5343	336 (6)	1492 (28)	305 (6)	3210 (60)	382	0 (0)	102 (26)	94 (25)	186 (49)
Spring fed wells	4099	784 (19)	498 (12)	175 (4)	2642 (64)	519	30 (6)	200 (39)	27 (5)	262 (50)
Brick & concrete pits	5511	49 (1)	3400 (62)	29 (1)	2033 (37)	156	0 (0)	66 (42)	1 (1)	89 (57)
Rice fields	4337	199 (5)	1956 (45)	347 (8)	1835 (42)	53	0 (0)	0 (0)	53 (100)	0 (0)
Tire trucks	1533	24 (2)	841 (55)	33 (2)	635 (41)	12	0 (0)	0 (0)	12 (100)	0 (0)
Hoofprints	22	0 (0)	10 (45)	1 (5)	11 (50)	0	0 (0)	0 (0)	0 (0)	0 (0)
Totals	56,058	11,058	17,942	4761	22,297	13,183	4840	2067	1197	5,079

### Seasonal variation in proportion of habitats inhabited by *An. funestus* (larval positivity)

In the dry season, 44.5% of aquatic habitats were positive for. *An. funestus;* with the highest occupancy in river streams (69%). Positivity rates were intermediate in ground pools and ditches (42% and 60% respectively) and minimal to null in other habitat types (Table 2). Overall positivity rates decreased to 24.5% in the rainy season; likely reflecting the increase in available habitats rather than a reduction in the *An. funestus* population. Stream segments, ground pools and ditches continued to have highest positivity as in the dry season (Table 2). However, spring-fed wells, brick/ concrete pits and rice field habitats that had had low or no *An. funestus* in the dry season showed higher positivity in the wet season (Table 2). Notably, the positivity of spring-fed wells for *An. funestus* increased from 5 to 34% between the dry and wet season.

**Fig. 2:**
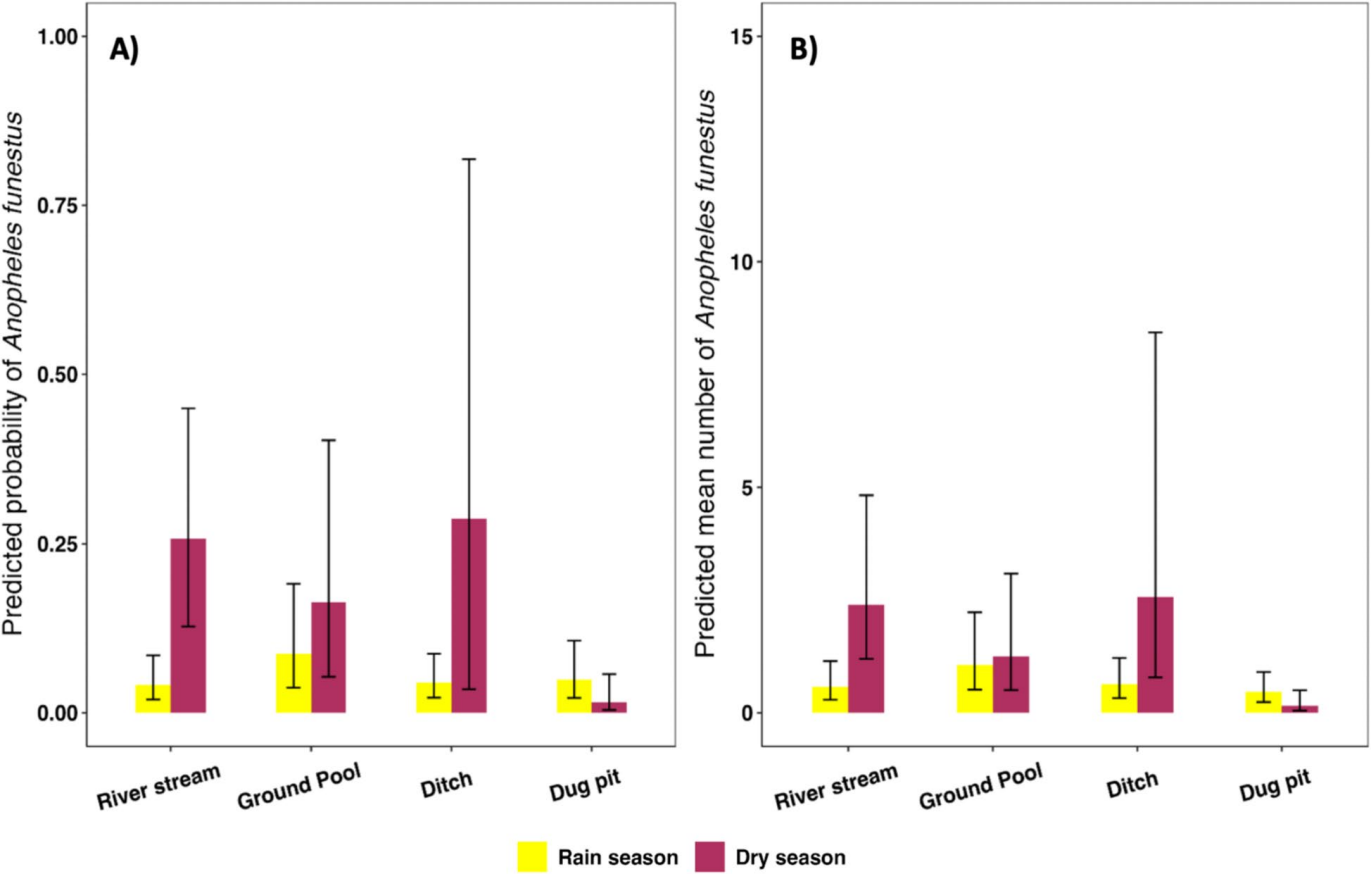
Seasonal comparison **A** *An. funestus* positivity, **B** *An. funestus* mean abundance. Bar plots show the predicted probabilities and mean for each habitat type during the rain and dry seasons, with error bars representing 95% confidence intervals. The statistical analysis was restricted only to 4 main habitat categories out of 9, where sample sizes were large enough to permit robust analysis in both seasons

**Fig. 3:**
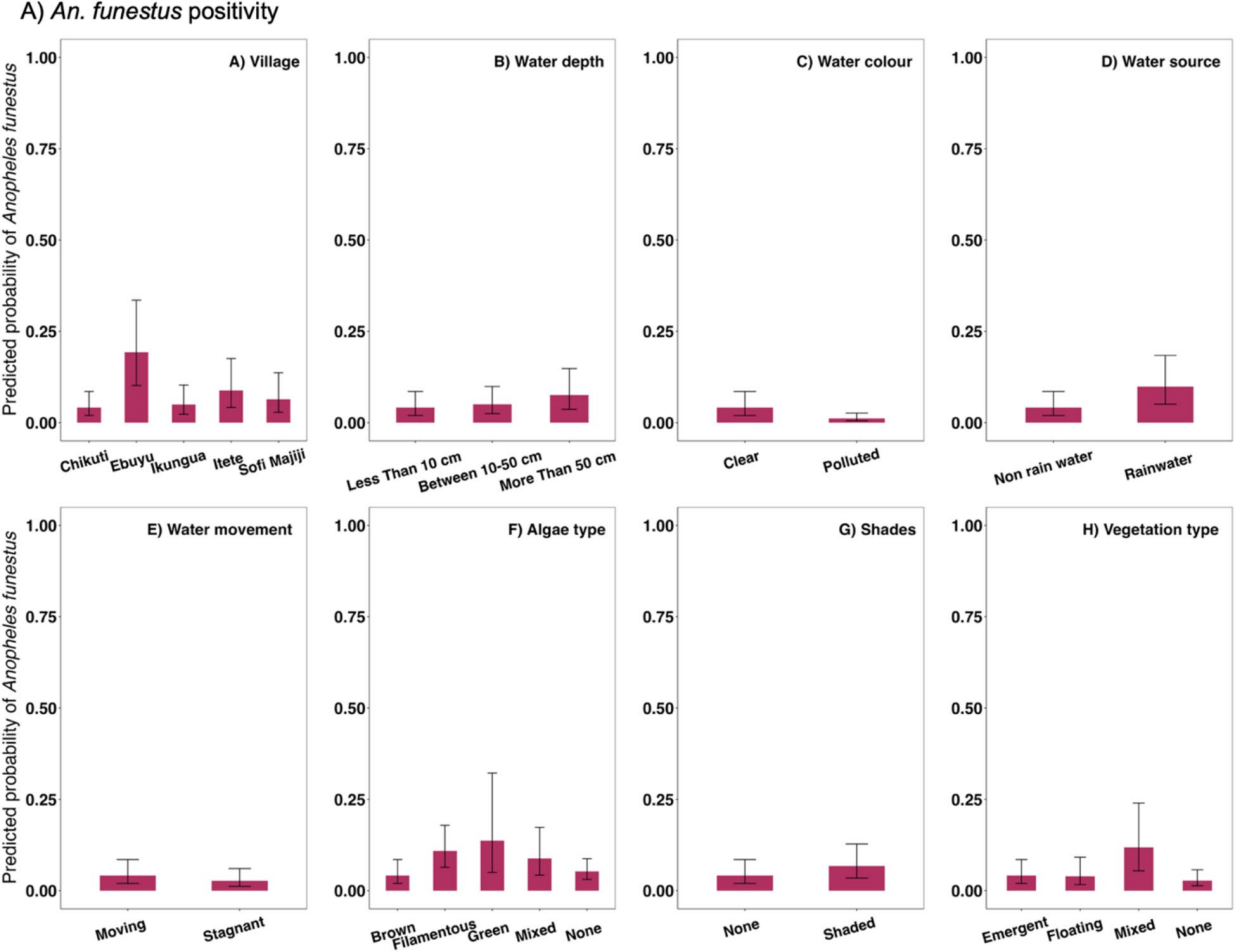
Predicted probability of *Anopheles funestus* positivity as influenced by environmental and habitat-related factors. The bar plots display predicted probabilities from a generalized linear mixed model analysis, with error bars indicating 95% confidence intervals

**Fig. 4:**
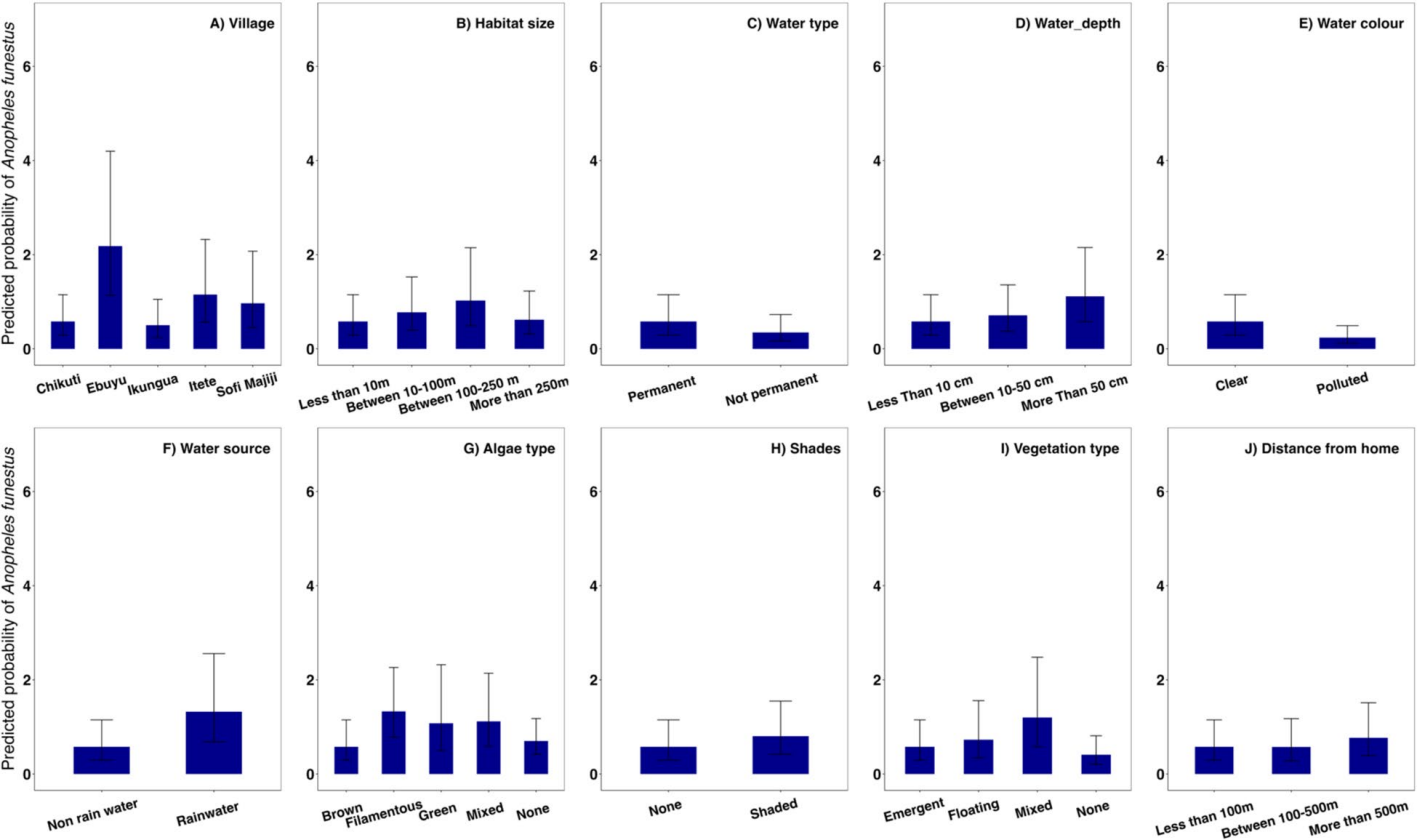
Mean predicted probability of *Anopheles funestus* abundance as influenced by environmental and habitat-related factors. The bar plots display predicted probabilities from a generalized linear mixed model analysis, with error bars indicating 95% confidence intervals

Of the 15 explanatory models considered in the GLMM, 11 showed a significant association with *An. funestus* positivity (SOM Table 3). Positivity was generally higher in the dry than wet season in most habitat types. However, the impact of season on *An. funestus* positivity varied between habitat types (habitat type*season interaction:*X*^*2*^ = 46.19, p < 0.001). Notably, positivity was higher in the dry than wet season in natural habitats such as streams, ground pools, and ditches, whereas positivity increased in the wet season in human-made habitats such as dug pits and spring fed wells, (Fig. 2A). There was also significant variation in *An. funestus* positivity between villages, with positivity highest in Ebuyu (z = 1.689, p < 0.001) and lowest in Chikuti (Fig. 3A). Environmental factors played an important role, with positivity being higher in habitats with deep water (more than 50 cm, z = 0.625, p < 0.005 Fig. 3B), that were rainwater fed (z = 0.925, p < 0.001, Fig. 3D), shaded (z = 0.925, p < 0.001), had filamentous algae (z = 3.484, p < 0.001) (SOM Table 4, Fig. 3A), and contained mixed vegetation (z = 5.299, p < 0.001) (SOM Table 1, Fig. 4A). In contrast, positivity was lower in habitats with visually polluted (z = -7.508, p < 0.001); Fig. 3A) and stagnant water (z = − 2.32, p = 0.02; SOM Table 4, Fig. 3A).

### Seasonal variation in abundance of An. funestus larvae

The mean abundance per dip in different habitats ranged from 0 to 2.3 and was generally higher in the dry than wet season, with the exception of spring-fed wells (Table 2). The total number of larvae collected was however greater in the wet season due to the overall greater number of habitats. Of the 14 explanatory variables tested, 11 were retained as statistically significant in the final model, indicating a robust association with *An. funestus* abundance (SOM Table 5). Mean larval abundance was significantly associated with an interaction between season and habitat type (SOM Table 6, Fig. 2B). This interaction showed that the effect of season on larval abundance varied depending on habitat type. For example, higher larval abundance was observed in river streams, ground pools, and ditches during the dry season, while in spring-fed wells, abundance was higher in the wet season. This pattern was similar to what was found for larval positivity, indicating that seasonal effects on larval production were influenced by habitat type.

**Fig. 5:**
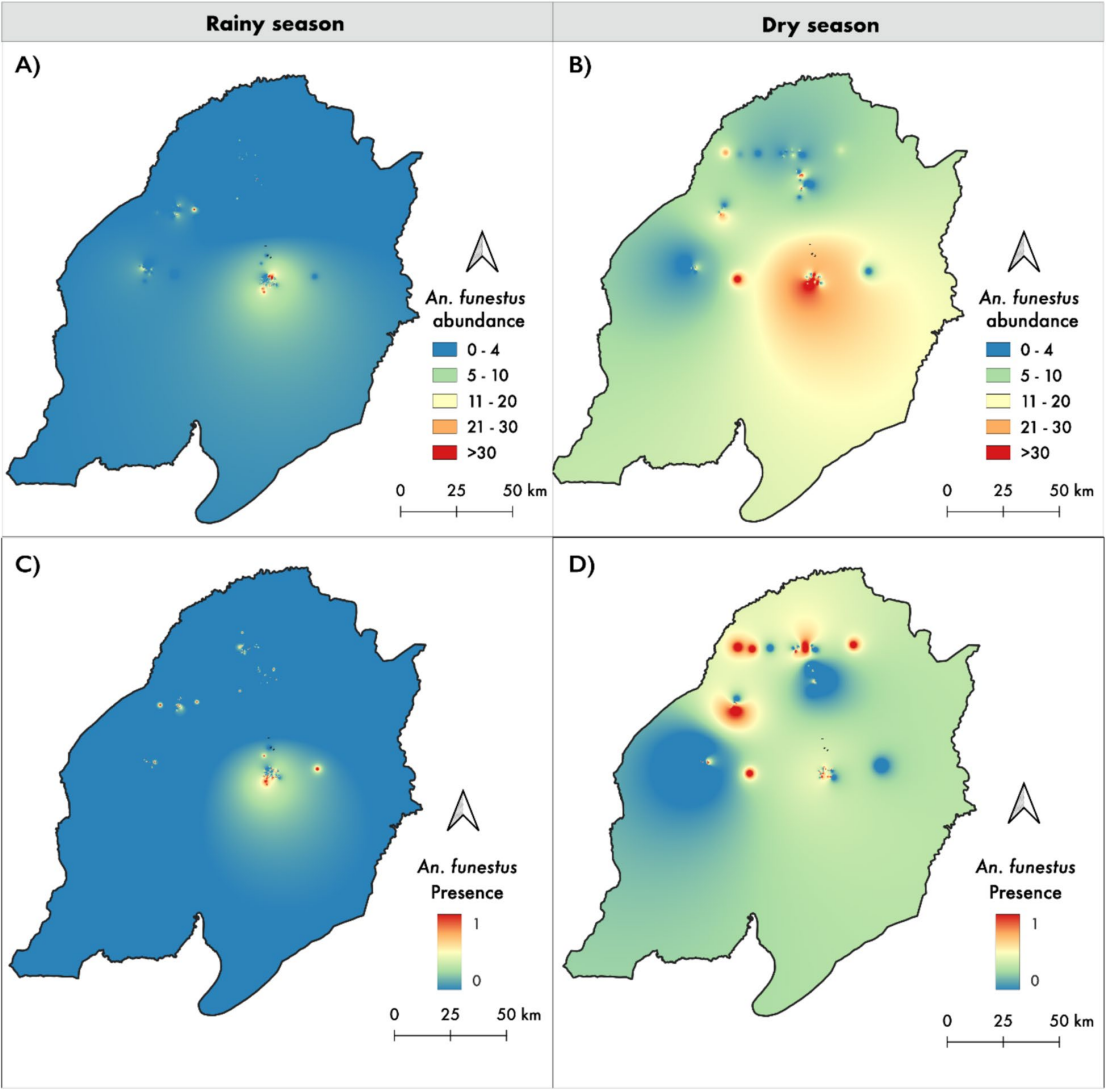
Inverse Distance Weighting (IDW) Maps of *An. funestus* Distribution across seasons. The top panels show the presence of *An. funestus* habitats during the rainy (**A**) and dry (**B**) seasons in the Ulanga and Malinyi district, with hotspots indicating a higher likelihood of habitat occurrence. The bottom panels illustrate the abundance of *An. funestus* larvae within identified habitats for the rainy (**C**) and dry (**D**) seasons, with warmer colors denoting higher larval counts

Mean larval abundance also varied significantly between villages, with Ebuyu recording the highest abundance (z = 10.422, p < 0.001, Fig. 5A), followed by Itete (z = 4.12, p < 0.001). Abundance was greatest in medium-sized (z = 2.734, p = 0.0062), permanent (z = 2.48, p = 0.013), and deep habitats (z = 3.83, p < 0.001), particularly those fed by rainwater (z = 6.20, p < 0.001), shaded (z = 3.27, p = 0.001), and containing filamentous algae (z = 3.41, p < 0.001). Conversely, mean abundance was significantly lower in polluted habitats (z = –6.87, p < 0.001), and higher in habitats surrounded by mixed vegetation (z = 5.43, p < 0.001) (Fig. 4).

### Seasonal variation in the spatial distribution of An. funestus habitats.

During the wet season, relatively low spatial heterogeneity was observed, with *An. funestus* distributed more uniformly across a higher number of habitats, although with lower mean larval abundance. The Moran’s I statistic for *An. funestus*positive habitats was 0.289 (z-score = 11.219, p-value < 0.001), indicating significant spatial autocorrelation. The IDW map for the wet season (Fig. 5A) showed a widespread but concentrated presence of larval sites, with a primary hotspot located in the central part of the study area.

In the dry season, the total number of larval habitats was reduced, but higher abundance was recorded within them. The distribution was more spatially heterogeneous, as shown by a higher Moran's I value of 0.411 (z-score = 5.95, p-value < 0.001), reflecting stronger spatial clustering of larval habitats (Fig. 5B). A major hotspot was observed in the same central area as in the wet season, with additional secondary clusters to the west and north.

The IDW maps for larval presence closely matched those for larval abundance (Fig. 5C and D), indicating that areas with a higher likelihood of larval occurrence also had higher larval densities.

## Discussion

This study tested whether the larval habitat use of the major African malaria vector *An. funestus* shifts seasonally in response to variation in aquatic habitat availability and type. Our findings challenge the paradigm that this species is relatively fixed in its use of larger and more permanent aquatic habitats; indicating a much higher than previously described adaptability for exploitation of different larval habitat as they become more available during the year. This reveals a high resilience to environmental variation in this species, which may account for its unique ability to sustain year-round malaria transmission in highly seasonal settings in Africa. This adaptability also poses challenges for the development of effective Larval Source Management strategies for this species, highlighting the need to cover a wider range of aquatic habitats for population targeting.

While it is widely acknowledged that there is large seasonal variation in availability of larval habitats for African malaria vectors due to rainfall [10, 16], we uniquely quantifying its magnitude In our study in southern Tanzania, remote sensing data indicated a 40% contraction in the area of land covered by water between the wet and dry season, with the total number of aquatic habitats identified falling by sevenfold (~ 2400 to ~ 300). These results align with broader trends observed in studies of seasonal changes in aquatic environments [10, 16]. We found that both the number of aquatic habitats and their diversity increased during the wet season; with a greater range of smaller, more temporary habitats becoming available during the rains. In the dry season, *An. funestus* were predominantly found in river streams, aligning with their previously described affinity for relatively permanent aquatic habitats. However, larval habitat use expanded in the rainy season to include a wider range of habitats, including those typically considered rare or unsuitable for *An. funestus* [6] (e.g., rice fields, dug pits and even tire tracks) This confirms that the larval habitat use of this species is seasonally variable, although most larvae were found in the same ‘top 3’ habitat types (river streams, ground pools, and ditches) in both seasons.

Our finding of higher aquatic habitat uses (% positivity) during the dry season matches observations across sub-Saharan Africa [28, 35, 39, 48], where *An. funestus* larvae are known to be more prevalent in drier periods [20]. This contrasts with the other major African malaria vector group *An. gambiae* sl, which is generally more prevalent and abundant during wetter months [30, 34]. The decrease in *An. funestus* larvae (both presence and abundance) during the rainy season may be due to habitat flooding which disperses larvae of this species from habitats, and/or the proliferation of aquatic habitats during the wet season outpacing the rate at which *An. funestus* can use them [5].

Beyond seasonal and habitat-type effects, several environmental factors significantly influenced *An. funestus* larval habitat use and abundance. In general, environmental factors that had a significant impact on larval positivity were similarly associated with abundance (8 out of 10 variables associated with abundance were also associated with positivity). Both larval outcome variables were positively associated with deeper, clear, and flowing aquatic habitats, habitats fed by rainwater, shaded, and surrounded by mixed vegetation, and that had green/filamentous algae (compared to no or brown algae). Many of these findings are in line with previous research on *An. funestus* larval ecology. For example, studies in western Kenya and south-eastern Tanzania found that the occurrence and abundance of *An. funestus* larvae was reduced in turbid waters [8, 31, 36, 37]. However, *An. funestus* larvae have been observed in turbid water in temporary natural habitats in Ethiopia [23]. Studies also reported that *An. funestus* were more likely to be found permanent, medium to large habitats (ranging from 10 to 250 square meters in surface area), and in habitats with emergent vegetation [20, 36, 37]; with similar effects for physio-chemical variables as detected here. Our finding of a positive association between *An. funestus* larvae, filamentous and green algae, and mixed vegetation contrast with an earlier study by Kahamba et al. [20] conducted in the dry season, where algae was negatively associated with the occurrence of *An. funestus* larvae [20]; suggesting seasonal variation in the importance and nature of environmental predictors of larval habitat use.

In addition to seasonal changes in *An. funestus* larval habitat use, the spatial distribution and clustering of their habitats also changed between the wet and dry season. There was spatial autocorrelation and clustering in the distribution of *An. funestus* habitats in both seasons, however this was more pronounced during the dry season. This can be explained by the contraction of habitats during the dry season, resulting in fewer and more spatially distinct clusters. This indicates LSM could be more effectively targeted and less expensive to implement during the dry season (with fewer habitats to target), assuming these persisting seasonal habitat hotspots are easy to find. A similar pattern of positive spatial autocorrelation in adult *An. funestus* has been documented in studies in Kenya [22] and Uganda [47]. Across both seasons, a substantial hotspot of *An. funestus* larval habitats was detected the center of the study, indicating a relatively fixed location of ‘high’ risk areas.

In combination, our findings have numerous implications for guiding LSM strategies for malaria in rural Africa, particularly in areas where *An. funestus* is a leading major vector species [33]. While this species’ usage of permanent habitats during the drier months provides an opportunity for highly targeted and effective LSM efforts, its expansion into a wider range of habitats including small and temporary sites during the wet season indicates such strategies need to be flexible and comprehensive [11]. Moreover, the large diversity and surface area of habitats in the wet season indicates that achieving high LSM coverage is unlikely to be feasible during this season. Instead, temporal targeting of LSM to during or near the end of the dry season may be more impactful both from a logistical and effectiveness perspective [9]. Historically, LSM for malaria vectors has been considered infeasible for many parts of rural Africa due to the proliferation of temporary habitats that would require treatment, especially during the rainy season. Dry season targeting may allow improved identification of key hotspots responsible for *An. funestus* production are identified, enabling LSM to be more effectively targeted [49].

This study had some limitations. Data collection was limited to two cross-sectional seasonal surveys, which may not capture finer-scale or transitional dynamics in larval habitat use throughout the year. Additionally, potential interannual variation in rainfall patterns and mosquito behavior could not be assessed (e.g. [4, 17]. Future studies should consider longitudinal designs that span multiple seasons and years to deepen ecological understanding and inform more adaptive LSM strategies under variable environmental conditions.

## Conclusions

This study provides clear evidence that larval habitat use by *Anopheles funestus*, a vector traditionally assumed to rely on fixed, permanent aquatic sites, is seasonally variable in response to rainfall. Contrary to the long-standing paradigm, *An. funestus* exhibits ecological adaptability by exploiting a broader range of aquatic habitats during the wet season, while concentrating in more stable habitats such as river streams and ground pools during the dry season.

Through extensive cross-sectional field surveys in south-eastern Tanzania, integrated with satellite image data and statistical modelling, we demonstrate that *An. funestus* is not restricted to permanent habitats but can also occupy temporary and human-made sites under favorable environmental conditions. This seasonal plasticity has important implications for LSM, suggesting that interventions should be tailored to target persistent habitats during the dry season and be flexible enough to include more diverse habitats in the rainy season. These findings reinforce the need for ongoing larval surveillance to inform spatial and temporal targeting of LSM, particularly in regions of east and southern Africa where *An. funestus* is a dominant malaria vector.

## Supplementary Information

Supplementary Material 1.

## Data Availability

All data for this study will be available upon reasonable request.
