# Supplementary material for: Seasonal variation in aquatic habitat availability and use by the malaria vector, Anopheles funestus
Source: Malar J. 2026 Apr 11;25:213. doi: 10.1186/s12936-026-05898-w (PMC13185408; doi:10.1186/s12936-026-05898-w)
Supplement: Supplementary file 1 — Supplementary Material 1. [file 12936_2026_5898_MOESM1_ESM.docx]

**Supplementary Material/ Appendix**

**Table 1:** Categories used to classify aquatic habitat types and physio-chemical characteristics.

| Feature Category | Description | Measurement Method |
| --- | --- | --- |
| Habitat Type  (category) | River streams (including stream pools formed after the evaporation), ground pools (such as marshes, swamps, and ponds), ditches (found within rice fields, beside rivers, and alongside roads), rice fields, spring-fed wells (comprising dug holes for spring water), pits (including brick pits and construction pits), puddles, hoofprints, and tire tracks | Visual observation |
| Water Movement | Slow, fast (moving), Stagnant | Visual observation, using dry stick |
| Water clarity | Clear, coloured (non-polluted), polluted | Visual observation |
| Water Source | Rainwater (surface water), non-rainwater (groundwater) | Local knowledge, visual observation |
| Water Permanence | Permanent- have water all year and historical stability across multiple seasons,  Semi-permanent - ones fluctuate but mostly retain water at least six months in a year,  Temporary - only have water temporarily mostly occurs during wet seasons, | Local knowledge, visual observation |
| Presence of vegetation | None or vegetated | Visual observation |
| Vegetation type | None, floating, emergent | Visual observation |
| Presence of algae | None or present | Visual observation |
| Algae type | Filamentous, green, blue-green, brown | Visual observation |
| Water Depth | Measured in cm, < 10 cm, 10-50 cm, > 50 cm | Long stick and measuring tape |
| Habitat Size, surface area | Measured in square meters, < 10 m², 10-100 m², 100-250 m², > 250 m² | GPS, measuring tape |
| Shade/canopy | Partial, heavily/fully (shaded), none | Visual observation |
| Surrounding Environment | Scrub/bush, cattle grazing, cultivated fields, residential area | Visual observation |
| Distance to the nearest house | Measured in square meters, < 100 m, 100-500 m, > 500 m | GPS |


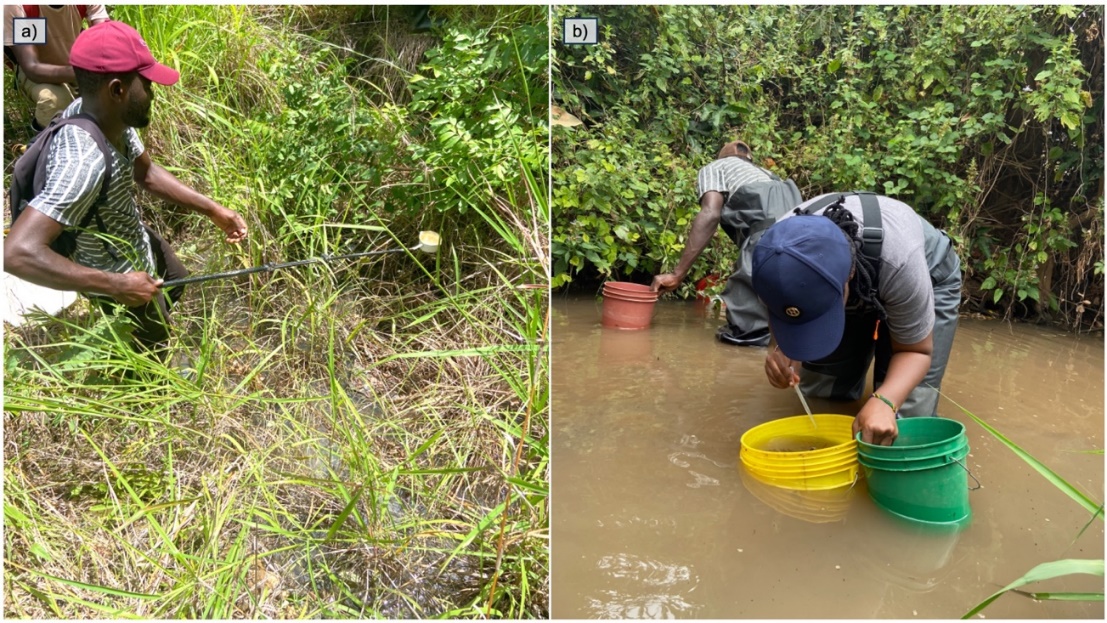


**Figure 1:** sampling techniques, a.) standard dipping b.) dipping using 10l bucket.

**Table 2:** Summary of Statistical Models used to include the primary response variable, explanatory variables, random effect variables, and statistical distribution used.

| **Model** | **Response variables** | **Fixed Effect variables** | **Random effect**  **variables** | **Statistical distribution** |
| --- | --- | --- | --- | --- |
| 1 | Availability of aquatic habitats (water and non-water classes) | Multispectral images with 8 bands: band 1 (coastal blue), band 2 (blue), band 3 (green i), band 4 (green), band 5 (yellow), band 6 (red), band 7 (red edge), band 8 (near-ir) | NA | Binomial distribution for binary classification |
| 2 | Presence or absence of *An. funestus* (positivity) | Season + Habitat type + Village + Habitat size + Water permanence + Water depth + Watercolor + Water source + Water movement + Algae type + Presence of shades + Vegetation + Distance from home + Habitat type: Season | Date | Binomial |
| 3 | Densities of *An. funestus* larvae (abundance) | Season + Habitat type + Village + Habitat size + Water permanence + Water depth + Watercolor + Water source + Water movement + Algae type + Presence of shades + Vegetation + Distance from home + Habitat type: Season | Date | Negative binomial distribution with zero inflation |

Model 1 is a classification tree model. For model 2 and 3, an offset term based on the volume of the water sample was included to account for variations in sampling effort, “**:**” indicates an “interaction”.

**Table 3:** Summary of stepwise model reduction for predicting *An. funestus* presence. The table shows the chi-squared statistics, degrees of freedom (d.f.), and p-values for the likelihood ratio test of each variable.

| **Deleted Variable** | **Chi-Sq** | **d.f.** | **P** |
| --- | --- | --- | --- |
| Habitat size | 4.52 | 3 | 0.2108 |
| Season * Village | 8.91 | 4 | 0.0633 |
| Distance from home | 5.72 | 2 | 0.0573 |
| Water type | 3.42 | 1 | 0.0644 |
| **Included variables** |  |  |  |
| Season | 116.98 | 4 | <0.001 |
| Habitat type | 58.34 | 6 | <0.001 |
| Village | 147 | 4 | <0.001 |
| Water depth | 12.09 | 2 | 0.0023 |
| Water colour | 66.01 | 1 | <0.001 |
| Water source | 38.39 | 1 | <0.001 |
| Water movement | 5.66 | 1 | 0.0173 |
| Algae type | 43.63 | 4 | <0.001 |
| Shades | 15.72 | 1 | <0.001 |
| Vegetation type | 57.49 | 3 | <0.001 |
| Habitat type: Season | 49.62 | 3 | <0.001 |

**Table 4:** Summary of the coefficient estimate (β), standard errors, z values and p-value for each explanatory variable included in the logistic model for predicting the presence of *An. funestus* aquatic habitats.

|  | **Variable** | **β** | **Std error** | **z value** | **p-value** |
| --- | --- | --- | --- | --- | --- |
|  | Intercept | -3.11 | 0.39 | -7.92 | <0.001 |
| Season: Habitat type | Dry season: Ditch | 0.06 | 1.21 | 0.06 | 0.95 |
|  | Dry season: Ground Pool | -1.35 | 0.55 | -2.44 | 0.015 |
|  | Dry season: Dug pits | -3.23 | 0.58 | -5.51 | <0.001 |
| Village | Ebuyu | 1.69 | 0.16 | 10.67 | <0.001 |
|  | Ikungua | 0.17 | 0.16 | 1.07 | 0.28 |
|  | Itete | 0.78 | 0.20 | 3.84 | <0.001 |
|  | Sofi Majiji | 0.44 | 0.22 | 1.98 | 0.05 |
| Water depth | Between 10-50 cm | 0.19 | 0.18 | 1.06 | 0.28 |
|  | More Than 50 cm | 0.62 | 0.22 | 2.78 | 0.005 |
| Water colour | Polluted | -1.29 | 0.17 | -7.51 | <0.001 |
| Water source | Rainwater | 0.92 | 0.16 | 5.88 | <0.001 |
| Water movement | Stagnant | -0.45 | 0.19 | -2.32 | 0.02 |
| Algae type | Filamentous | 1.02 | 0.29 | 3.48 | <0.001 |
|  | Green | 1.28 | 0.56 | 2.29 | 0.0221 |
|  | Mixed | 0.79 | 0.39 | 2.03 | 0.042 |
|  | None | 0.24 | 0.29 | 0.85 | 0.39 |
| Shades | Shaded | 0.506 | 0.13 | 3.84 | <0.001 |
| Vegetation type | Floating | -0.05 | 0.27 | -0.21 | 0.83 |
|  | Mixed | 1.12 | 0.21 | 5.29 | <0.001 |
|  | None | -0.42 | 0.12 | -3.36 | <0.001 |

**Table 5:** Summary of stepwise model reduction for predicting *An. funestus* abundance. The table shows the chi-squared statistics, degrees of freedom (d.f.), and p-values for the likelihood ratio test of each variable.

| **Variables** | **X^2^** | **df** | **p-value** |
| --- | --- | --- | --- |
| Season | 106.69 | 32 | <0.001 |
| Habitat type | 67.76 | 26 | <0.001 |
| Village | 172.88 | 28 | <0.001 |
| Habitat size | 12.05 | 29 | 0.007 |
| Water type | 6.71 | 31 | 0.009 |
| Distance from home | 11.09 | 30 | 0.003 |
| Shades | 11.18 | 31 | 0.0008 |
| Water depth | 23.96 | 30 | <0.001 |
| Water source | 44.12 | 31 | <0.001 |
| Algae type | 51.46 | 28 | <0.001 |
| Water colour | 51.78 | 31 | <0.001 |
| Vegetation type | 60.64 | 29 | <0.001 |
| Habitat type: Season | 46.19 | 29 | <0.001 |

**Table 6:** Regression Coefficients from GLMM Model predicting *An. funestus* abundance.

|  | **Variable** | **β** | **Std. Error** | **z value** | **p-value** |
| --- | --- | --- | --- | --- | --- |
|  | Intercept | -0.76 | 0.37 | -2.06 | 0.04 * |
| Season: Habitat type | Dry season: Ditch | -0.02 | 0.53 | -0.03 | 0.96 |
|  | Dry season: Ground Pool | -1.26 | 0.36 | -3.44 | <0.001 |
|  | Dry season: Dug pits | -2.52 | 0.53 | -4.70 | <0.001 |
| Village | Ebuyu | 1.32 | 0.13 | 10.42 | <0.001 |
|  | Ikungua | -0.14 | 0.16 | -0.91 | 0.36 |
|  | Itete | 0.68 | 0.17 | 4.11 | <0.001 |
|  | Sofi Majiji | 0.51 | 0.19 | 2.60 | 0.009 ** |
| Habitat size | Between 10 - 100 m | 0.29 | 0.13 | 2.26 | 0.02 * |
|  | Between 100-250 m | 0.57 | 0.21 | 2.73 | 0.006 ** |
|  | More than 250m | 0.06 | 0.14 | 0.43 | 0.66 |
| Water type | Permanent | 0.51 | 0.20 | 2.47 | 0.01 * |
| Water depth | Between 10-50 cm | 0.20 | 0.14 | 1.38 | 0.16 |
|  | More Than 50 cm | 0.65 | 0.17 | 3.82 | <0.001 |
| Water colour | Polluted | -0.88 | 0.12 | -6.87 | <0.001 |
| Water source | Rainwater | 0.83 | 0.13 | 6.20 | <0.001 |
| Algae type | Filamentous | 0.83 | 0.24 | 3.41 | <0.001 |
|  | Green | 0.62 | 0.37 | 1.68 | 0.09. |
|  | Mixed | 0.65 | 0.29 | 2.24 | 0.02 * |
|  | None | 0.19 | 0.24 | 0.80 | 0.42 |
| Shades | Shaded | 0.33 | 0.10 | 3.27 | 0.001 ** |
| Vegetation type | Floating | 0.23 | 0.19 | 1.18 | 0.23 |
|  | Mixed | 0.73 | 0.13 | 5.43 | <0.001 |
|  | None | -0.34 | 0.09 | -3.67 | <0.001 |
| Distance from home | Between 100-500m | -0.01 | 0.15 | -0.07 | 0.94 |
|  | More than 500m | 0.28 | 0.09 | 3.14 | <0.001 |

**NB:** Dug pits included spring fed wells and brick & concrete pits, rice field, tire track and hoofprints were removed from this analysis since they were only available in rainy season only
